# Sublethal Exposure to Phosphine Decreases Offspring Production in Strongly Phosphine Resistant Female Red Flour Beetles, *Tribolium castaneum* (Herbst)

**DOI:** 10.1371/journal.pone.0053356

**Published:** 2012-12-31

**Authors:** Andrew W. Ridley, Seymour Magabe, David I. Schlipalius, Michelle A. Rafter, Patrick J. Collins

**Affiliations:** 1 Agri-Science Queensland, Department of Agriculture, Fisheries and Forestry, EcoSciences Precinct, Brisbane, Queensland, Australia; 2 Cooperative Research Centre for National Plant Biosecurity, Bruce, Australian Capital Territory, Australia; 3 Perum BULOG, Jakarta, Indonesia; 4 Department of Agricultural Sciences, La Trobe University, Bundoora, Victoria, Australia; United States Department of Agriculture, Agriculture Research Service, United States of America

## Abstract

The red flour beetle is a cosmopolitan pest of stored grain and stored grain products. The pest has developed resistance to phosphine, the primary chemical used for its control. The reproductive output of survivors from a phosphine treatment is an important element of resistance development but experimental data are lacking. We exposed mated resistant female beetles to 0.135 mg/L of phosphine for 48 h at 25°C. Following one week of recovery we provided two non-exposed males to half of the phosphine exposed females and to half of the non-exposed control females. Females that had been exposed produced significantly fewer offspring than non-exposed females. Females that remained isolated produced significantly fewer offspring than both exposed females with access to males and non-exposed controls (*P*<0.05). Some females were permanently damaged from exposure to phosphine and did not reproduce even when given access to males. We also examined the additional effects of starvation prior to phosphine exposure on offspring production. Non-exposed starved females experienced a small reduction in mean offspring production in the week following starvation, followed by a recovery in the second week. Females that were starved and exposed to phosphine demonstrated a very significant reduction in offspring production in the first week following exposure which remained significantly lower than that of starved non-exposed females (*P*<0.05). These results demonstrate a clear sublethal effect of phosphine acting on the female reproductive system and in some individuals this can lead to permanent reproductive damage. Pest population rebound after a fumigation may be slower than expected which may reduce the rate of phosphine resistance development. The results presented strongly suggest that phosphine resistance models should include sublethal effects.

## Introduction

The red flour beetle, *Tribolium castaneum* (Herbst), is a cosmopolitan pest of stored grain, flour mills and grain products [1], [2], [3], and the fumigant phosphine is commonly used for its control. However, high level resistance to phosphine has been reported in most countries where it has been surveyed [4], [5], [6], [7], [8], [9]. Adults of this pest are long lived and single mating pairs have been recorded to produce greater than 500 offspring over a three month period [10], [11], [12].

The development of heritable resistance to pesticides by insects is an evolutionary process that is wholly dependent on the survivors of a selection event (i.e. pesticide application) producing offspring. This results in post-selection populations having a higher frequency of resistance alleles compared with pre-selection populations. In systems where resistance selection occurs on long-lived and fecund adults, sublethal effects on the ability of the adults to reproduce subsequent to a selection event may have a significant impact on the development of resistance.

A reduction in the number of eggs laid by fumigated females of a susceptible and a relatively weak (x 6), laboratory selected, phosphine-resistant strain of *T. castaneum* has been reported [13] and the observed reduction in fecundity was dose dependent. The fecundity of strongly phosphine resistant *Rhyzopertha dominica* (F.), measured as production of offspring, was temporarily reduced following a sublethal dose of phosphine, but full recovery occurred within two weeks [14]. *Tribolium castaneum* adults and a close relative *Tribolium confusum* du Val, regained tolerance of phosphine, measured by mortality, in four and ten days respectively, suggesting that full recovery is achieved by survivors of phosphine exposure [15], [16]. However, observations made on the cockroach *Periplaneta americana* (L.) suggest that, at least for that species, injury induced by phosphine exposure is irreversible [17]. The induction of observable irreversible symptoms in *P. americana* that affect behaviour, implies that some tissue-specific necrosis may have occurred (most likely in neural tissue), from which full recovery is not possible.

Reduced metabolism has been linked to resistance to phosphine [18], and the most metabolically active life stages of wild type beetles appear to be the least tolerant to phosphine [19], [20]. During periods of starvation, metabolism is reduced in the fly *Drosophila melanogaster*, [21], [22]. Therefore, beetles that have a period of reduced metabolism (during starvation for example) may have greater tolerance to the toxic effects of phosphine. *Tribolium castaneum* generally tolerates periods of starvation better than other stored product beetles [23], [24], [25], and this may be a result of an ability to reduce its metabolism to conserve energy.

We describe a laboratory study in which we test whether sublethal exposure to phosphine reduces reproduction in strongly resistant (x 431) *T. castaneum* females. To test if any sublethal effect is a result of sperm depletion or female injury we allow exposed females to re-mate with non-exposed males. We also examine the additional effects on females from a period of starvation prior to exposure.

## Methods

### Test Strain

Insects used in this study were from a strongly phosphine resistant strain of *T. castaneum* (QTC931) [9] reared on wholemeal flour+yeast (20∶1 w/w) at 30°C 60% r.h. This strain originated as a resistant field sample from southern Queensland in 2000 and had been selected in the laboratory to ensure homozygosity for resistance. Sexing of pupae and adults was as described by Hinton [26]. No specific permits were required for the described field studies. *Tribolium castaneum* is not an endangered or protected species.

### Phosphine Exposure Procedure

All exposures were carried out as described previously [14], at a dosage of 0.135 mg/L for 48 h at 25°C. Phosphine was generated from a commercial formulation of aluminium phosphide and collected over acidified distilled water [27]. The source concentrations were measured with a gas chromatograph using a thermal conductivity detector (Clarus 500, PerkinElmer). Adults were individually placed into 96-well flat-bottomed microtitre plates with filter paper covering the wells to prevent escape but allow phosphine penetration. The plates were placed into a desiccator and the measured dose of phosphine was injected through a rubber septum using a gas-tight syringe.

### Impact of Sublethal Exposure on Tribolium Castaneum Oviposition and the Impact of Access to Non-exposed Males

Five hundred randomly selected 1- to 2-week old adults of mixed sex were placed individually in polyurethane diet cups containing 10 g of culture medium for one week at 30°C and 55% r.h., at which point 300 were then dosed as above. The remaining 200 beetles were used as non-exposed controls and were placed in desiccators without phosphine. Following exposure, all beetles were placed into 29 ml volume polypropylene portion cups with 10 g of medium and transferred each week to fresh cups for a total of four weeks. From the beginning of the second week post exposure, half of the exposed and half of the non-exposed beetles were given access to two 1- to 2-week old non-exposed males. Beetle trios were transferred weekly thereafter. The gender of all beetles was determined at the end of the fourth week post exposure and any cup that had contained a single male or three males was discarded. Females that died during the four week post exposure period were not considered to have survived the fumigation so were not used in data analysis. The four experimental groups contained different numbers of females as a result of mortality caused by the fumigation. Data from 42 isolated non-exposed females, 32 non-exposed females with access to males, 25 isolated exposed females and 13 exposed females with access to males were used for analysis. Each female was considered a replicate. The plastic cups were maintained at 30°C and 55% r.h. for six weeks after beetles had been transferred at which point the adult offspring produced by experimental females were counted.

### Effect of Starvation Prior to Sublethal Exposure to Phosphine

Two hundred 1– to 2-week old adults were randomly selected from a culture maintained as described above. It was assumed that all females were mated. Beetles were placed individually into polyurethane diet cups containing 10 g of medium for one week and kept at 30°C and 55% r.h. The beetles were subsequently transferred to individual wells of a flat bottomed 96-well microtitre plate as described above. There were 50 beetles in each of four plates. The beetles were starved in the plates for one week at 25°C and 55% r.h., after which 100 individuals were exposed to phosphine as above. The other 100 beetles were treated as non-exposed controls and kept in desiccators at 25°C and 55%. After fumigation both exposed and control beetles were placed individually into diet cups with 10 g of medium and transferred each week to fresh cups for a total of four weeks after the exposure. Gender of test beetles was determined for all individuals at the end of week four post-exposure and males were excluded from analysis. Females that did not produce offspring in the pre-exposure and pre-starvation week were treated as unmated and were eliminated from further analysis. Females that died during the 4-week post-fumigation period were not considered to have survived the fumigation so were not used in data analysis. Data from 39 starved females and 31 starved and exposed females were used in the analysis. Each female was considered a replicate. The cups that contained female beetles were maintained at 30°C and 55% r.h. for a further six weeks and the emerged progeny were counted.

### Statistical Analysis

Statistical analyses were performed with the GenStat software [28]. A number of females once exposed to phosphine did not produce any offspring. This created a bimodal distribution of offspring production. A binomial analysis where females were categorised as reproductive or non-reproductive following exposure was conducted separately for both experiments. A generalised linear model with a binomial distribution and a logit link was fitted to the data. For the experiment assessing the effect of exposure and access to males, ‘access to males’, ‘exposure’ and the interaction was originally fitted to the model but neither the interaction nor the ‘access to males’ term were found to be significant so were dropped from the final model. Females exposed to phosphine were significantly more likely to be non-reproductive compared to females that were not exposed for both experiments, (d.f. = 1 deviance ratio 30.29 P<0.01) and (d.f. = 1 deviance ratio = 9.23, P = 0.002) for the access to males experiment and the starved prior to exposure experiment respectively. A goodness of fit test was performed and indicated a good fit to the data in both experiments. The non-reproductive females were then removed from the dataset to explore the effects of phosphine on the production of offspring in reproductive females.

The distribution of offspring counts were assessed using histograms. These graphs showed no significant deviation from a normal distribution except in the first week post exposure, and showed no indication that another distribution would better fit the data. Analyses on the number of offspring for reproductive females were performed using residual maximum likelihood (REML) methods. For both experiments ‘exposure’, ‘oviposition week’ and their interaction were fitted as fixed effects while ‘female’ by ‘oviposition week’ was fitted as a random effect to account for the repeated measures. The experiment which also assessed the effect of access to males included ‘access to males’ and all interactions with it as fixed effects. After assessing the correlations between time-points and testing several covariance structures, an auto-regressive structure of order 1 was fitted. Residual plots showed no significant deviations from model assumptions. All analyses were performed at the 5% significance level, with pair-wise comparisons being performed using Fisher’s Protected Least Significant Differences. Sex ratio data were analysed by Chi-square.

## Results

### Impact of Sublethal Exposure on Tribolium Castaneum Oviposition and the Impact of Access to Non-exposed Males

The total mortality for all exposed individuals was 64.1% and 2.1% for non-exposed individuals. All of the non-exposed females that had access to males produced offspring in all of the oviposition weeks of the experiment. Forty-one of the forty-two isolated females (98%) that were not exposed produced offspring in the four weeks after the exposure period. This is in contrast to the nine out of thirteen (69.2%) and fourteen out of twenty-five (56%) exposed females with access to males and isolated females, respectively, that produced offspring following exposure. Access to males was not found to significantly increase the likelihood of a female being reproductive following exposure to phosphine (d.f. = 1, deviance ratio = 1.10, P = 0.295). Of the reproductive females, there was a significant interaction between the effects of exposure, whether females had access to males and oviposition week (F = 4.66, d.d.f. = 363.5, P = 0.001). Although all females produced relatively high numbers of offspring prior to phosphine exposure, significantly fewer offspring were produced by females that would go on to be fumigated and then have access to males compared to the other groups (Table 1). The non-exposed females continued to produce relatively large numbers of offspring for the four weeks of oviposition. Non-exposed females that had access to males produced more offspring on average than isolated females but this increase was not statistically significant over the course of the experiment. Exposed females produced significantly fewer offspring in the first week post-exposure compared to non-exposed females. The mean fecundity of isolated exposed females increased during the second week after exposure but was significantly lower than the fecundity of non-exposed females in all weeks after exposure. Exposed females with access to males produced significantly more offspring on average than exposed females that did not and during weeks three and four post exposure produced statistically similar numbers of offspring as the non-exposed females (Table 1). Table 1 displays the means offspring production for reproductive females only. To obtain mean offspring production for all surviving females in a treatment multiply this mean by the proportion of reproductive females.

**Table 1:** Mean offspring production by non-exposed and exposed mated females that were isolated or given access to non-exposed males.

Week	Non-Exposed ♀♀		Exposed ♀♀	
	♀♀ only	♀♀ plus ♂♂	♀♀ only	♀♀ plus ♂♂
Pre exposure	103.59^a,b^	99.66^b,c,d^	93.50^b,c,d,e^	79.78^e,f,g^
Post exposure Wk 1	91.85^c,d,e^	94.09^b,c,d,e^	7.36^j^	5.78^j^
Post exposure Wk 2	97.41^b,c,d^	114.00^a^	60.86^g,h^	73.22^f,g^
Post exposure Wk 3	98.46^b,c^	112.16^a^	50.57^h^	97.33^a,b,c,d,e^
Post exposure Wk 4	90.66^d,e^	101.56^b,c,d^	37.14^i^	90.56^b,c,d,e,f^
Standard errors	3.69	4.18	6.32	7.88

Females that did not reproduce following exposure to phosphine were excluded from the analysis (see methods). Standard errors are calculated based on the pooled variance for each column.

Rows and columns with the same superscript are not significantly different (P>0.05).

The sex ratio of offspring produced in the week prior to exposure (565 ♀ to 495 ♂) and in the first week after exposure (489 ♀ to 453 ♂) did not significantly deviate from unity (*X* ^2^ = 1.4; df = 1; P = 0.69).

### Effect Starvation Prior to Sublethal Exposure to Phosphine

The total mortality for exposed individuals following seven days of starvation was 33.3%. All mortality occurred in the two weeks immediately following exposure. Starved non-exposed individuals had 8.3% mortality over the course of the experiment. All mortality in this case also occurred in the first two weeks following starvation. Thirty-eight of the thirty-nine females (97.5%) from the starved non-exposed treatment produced some offspring in all four oviposition weeks following starvation. Twenty-three out of thirty-one (74.2%) starved and exposed females produced offspring in the four week period following treatment.

There was a significant interaction between exposure and oviposition week for those females that did produce offspring following exposure (F = 18.60, d.d.f. = 238.2, P<0.001). Fewer offspring were produced by females that were subsequently exposed, prior to starvation and exposure. This difference was small but statistically significant (Table 2). A significant reduction in the mean offspring production by non-exposed females occurred after seven days of starvation. There was a significant recovery of offspring production in the second week post starvation followed by a gradual decline in mean fecundity in weeks three and four (Table 2). Females that were starved and exposed to phosphine demonstrated a significant reduction in offspring production in all weeks following exposure compared to the non-exposed females. Offspring production of exposed females was at its lowest in the first week after exposure. The mean number of offspring produced by starved and exposed females increased significantly in weeks two to four compared to week one, but was always significantly lower than the mean number of offspring produced by non-exposed starved females (Table 2).

**Table 2:** Mean offspring production by non-exposed and exposed mated females starved for seven days prior fumigation.

Week	Starved/non-exposed♀♀	Starved/exposed♀♀
Pre starvation/exposure	90.15^a,b^	78.04^c,d^
Post exposure Wk 1	65.44^e,f^	8.17^g^
Post exposure Wk 2	96.74^a^	59.96^f^
Post exposure Wk 3	86.46^b,c^	70.13^d,e^
Post exposure Wk 4	76.95^d^	58.91^f^
Standard errors	3.67	4.78

Females that did not reproduce following exposure to phosphine were excluded from the analysis (see methods). Standard errors are calculated based on the pooled variance for each column.

Rows and columns with the same superscript are not significantly different (P>0.05).

The sex ratio of offspring produced in the week prior to exposure (697 ♀ to 706 ♂) and in the first week after exposure (433 ♀ to 490 ♂) did not significantly deviate from unity (*X* ^2^ = 3.4; df = 1; P = 0.333).

## Discussion

Sublethal exposure to phosphine decreased the production of offspring of strongly resistant *T. castaneum* females. This result confirms the sublethal effects of phosphine on fecundity reported by [13]. A majority of mated females produced offspring following a sublethal exposure to phosphine without having to re-mate. However, about 30% of the surviving females lost the ability to reproduce altogether following a sublethal exposure to phosphine even if given access to males. In the laboratory, *T. castaneum* adults mate frequently with multiple partners, and females can use stored sperm to produce offspring for several months [29], [30] Therefore, resistant females mated before a selection event would have enough stored sperm to potentially produce relatively high numbers of offspring after surviving a sublethal dose of phosphine even without re-mating. It is not known whether the non-fecund post-treatment females either did not mate with the males on offer or did mate but were unable to use the sperm provided, but all live females appeared to be behaviourally normal (i.e. ran when disturbed and burrowed into the culture medium). Females that were able to mate should have done so when placed with males in such a small volume of media. Therefore we hypothesise that phosphine exposure permanently damaged these females’ reproductive systems. *Tribolium castaneum*, like other Coleoptera Polyphaga, have telotrophic meroistic ovarioles in which all germ-cell proliferation and differentiation takes place during the larval and pupal stages [31]. A possible explanation for the permanent reduction or complete loss of offspring production observed in some females is that phosphine exposure killed or incapacitated a majority or all of the available oocytes. The exposed females have, therefore, fewer or no oocytes to fertilise and oviposit even when provided with fresh sperm. The loss of reproduction by some *T. castaneum* females contrasts with another study on *R. dominica* in which only one out of twenty-five fumigated females did not produce any offspring [14], and this female was considered, by the authors, not to be mated. These results suggest that at the applied dosages, phosphine causes more damage to the reproductive system of female *T. castaneum* than *R. dominica.*

Reduced oviposition as a result of insecticide exposure has been reported previously. Female *T. confusum* exposed to 50 ppm of DDT or 10 ppm lindane demonstrated a 70% reduction in oviposition [32]. There were no anatomical or histopathological changes observed in the reproductive organs of the DDT-treated females and the authors suggested that the reduction in oviposition was a result of retention or resorption of eggs. In these studies the experimental beetles were ovipositing into insecticide impregnated flour so retention of eggs as a response to chemical exposure is a more plausible explanation of the results than in our experiments where females were exposed for only a relatively short period and left to recover and oviposit in the absence of insecticide. A sublethal dose of methoprene almost eliminated offspring production in some *T. castaneum* individuals but only when the beetles were exposed as late-instar larvae [33].

Sublethal exposure to phosphine had the same overall effect on the offspring production of starved females as it did on non-starved females when comparison is made across the two experiments. A similar pattern of offspring production recovery was observed for the females starved before exposure as for females not starved. Starvation prior to exposure did not inhibit the toxic action of phosphine but the starved and exposed females produced more offspring compared to non-starved exposed females in the first experiment. Females in both experiments were exposed to the same dosage of phosphine and beetles that were not starved prior to fumigation suffered twice the mortality of the starved beetles. A direct test is needed, however, to clarify this point. Starvation for seven days reduced offspring production of non-exposed individuals in the week immediately following starvation. These results disagree with those of Daglish [25] who reported no reduction in offspring production following seven days of starvation. Daglish [25] used mixed sex batches of beetles whereas the females in our experiments were isolated from males following the starvation period which may explain the different results.

Non-exposed females that had access to males maintained a relatively constant rate of offspring production over the course of the experiment which supports previous studies by Howe [12], that oviposition by *T. castaneum* was maintained at a relatively constant rate for at least 100 days when single pairs of beetles were observed. In our experiments non-exposed females that did not have access to males produced fewer offspring but the reduction was not statistically significant. The effect of access to sperm via additional males was most significant in the fumigated treatments. Freshly mated females start laying eggs one day after mating and after two days lay just as many eggs as already mated females [34]. Therefore, if sperm depletion was responsible for the observed cessation of offspring production, the exposed females should have produced offspring again immediately after males were available. This delay in the recovery of offspring production suggests that sublethal exposure temporarily suppresses the female reproductive system as well as killing or incapacitating a significant amount of stored sperm.

The results presented here demonstrate a clear sublethal effect of phosphine on strongly resistant females in the form of reduced fecundity. Whether phosphine has a sublethal effect on male *T. castaneum* remains to be tested, but Ridley et al. [14] demonstrated a significant temporary reduction in offspring production when exposed males were mated with non-exposed females in *R. dominica*. The results presented here suggest that current models of phosphine resistance development which do not include any sublethal effects [35], [36], should be adjusted. The impact of the sublethal effects could then be assessed under different scenarios. The dosage used in our experiments was high enough to kill all susceptible individuals [9]. Therefore the rate of immigration of beetles with susceptible genes into the grain storage after the selection event may have a significant impact on the development of resistance. If immigration is negligible, the sublethal effects will only reduce population growth. If immigration is relatively high, the sublethal effects will aid in the dilution of the population with susceptible genes.

## References

[1] Arthur FH, Hagstrum DW, Flinn PW, Reed CR, Phillips TW (2006) Insect populations in grain residues associated with commercial Kansas grain elevators. Journal of Stored Products Research 42: 226–239.

[2] Campbell J, Toews MD, Arthur FH, Arbogast RT (2010) Long-term monitoring of *Tribolium castaneum* in two flour mills: seasonal patterns and impact of fumigation. Journal of Economic Entomology 103: 991–1001. 20568648 10.1603/ec09347

[3] White GG (1982) The effect of grain damage on development in wheat of *Tribolium castaneum* (Herbst) (*Coleoptera: Tenebrionidae*). Journal of Stored Products Research 18: 115–119.

[4] Price LA, Mills KA (1988) The toxicity of phosphine to the immature stages of resistant and susceptible strains of some common stored product beetles, and implications for their control. Journal of Stored Products Research 24: 51–59.

[5] Benhalima H, Chaudhry MQ, Mills KA, Price NR (2004) Phosphine resistance in stored-product insects collected from various grain storage facilities in Morocco. Journal of Stored Products Research 40: 241–249.

[6] Pimentel MAG, Faroni LRDA, Tótola MR, Guedes RNC (2007) Phosphine resistance, respiration rate and fitness consequences in stored-product insects. Pest Management Science 63: 876–881. 17597470 10.1002/ps.1416

[7] Taylor RWD, Halliday D (1986) The geographical spread of resistance to phosphine by Coleopterous pests of stored products; In British Crop Protection Conference – Pest and Diseasis, Brighton Metropole, England, November 17–20, 1986, Volume. 1: 607–613.

[8] Opit GP, Phillips TW, Aikins MJ, Hasan MM (2012) Phosphine resistance in *Tribolium castaneum* and *Rhyzopertha dominica* from stored wheat in Oklahoma. Journal of Economic Entomology 105: 1107–1114. 22928286 10.1603/ec12064

[9] Jagadeesan R, Collins PJ, Daglish GJ, Ebert PR, Schlipalius DI (2012) Phosphine resistance in the rust red flour beetle, *Tribolium castaneum* (Coleoptera: Tenebrionidae): inheritance, gene interactions and fitness costs. PLoS ONE 7. 10.1371/journal.pone.0031582 PMC3283673 22363681

[10] Simwat GS, Chalal BS (1975) Effect of the age of *Tribolium castaneum* Herbst (Coleoptera: Tenebrionidae) on the rate of egg laying and fecundity. Bulletin of Grain Technology 13: 23–25.

[11] Howe RW (1962) The effects of temperature and humidity on the oviposition rate of *Tribolium cataneum* (Herbst.) (Coleoptera, Tenebrionidae). Bulletin of Entomological Research 53: 301–310.

[12] Good NE (1933) Biology of the flour beetles, *Tribolium confusum* Duv. and *T. ferrugineum* Fab. Journal of Agricultural Research 46: 327–334.

[13] Saxena JD, Bhatia SK (1980) Reduction in fecundity of *Tribolium castaneum* due to fumigation and phosphine resistance. Indian Journal of Enomology 42: 796–798.

[14] Ridley AW, Schlipalius DI, Daglish GJ (2012) Reproduction of phosphine resistant *Rhyzopertha dominica* (F.) following sublethal exposure to phosphine. Journal of Stored Products Research 48: 106–110.

[15] Hobbs SK, Bond EJ (1989) Response of *Tribolium castaneum* (Herbst) (Coleoptera:Tenebrionidae) to sublethal treatments with phosphine. Journal of Stored Products Research 25: 137–146.

[16] Bond EJ, Upitis E (1973) Response of three insects to sublethal doses of phosphine. Journal of Stored Products Research 8: 307–313.

[17] Bond EJ, Robinson JR, Buckland CT (1969) The toxic action of phosphine - absorption and symptoms of poisoning in insects. Journal of Stored Products Research 5: 289–298.

[18] Pimentel MAG, Faroni LRDA, Totola MR, Guedes RNC (2007) Phosphine resistance, respiration rate and fitness consequences in stored-product insects. Pest Management Science 63: 876–881. 17597470 10.1002/ps.1416

[19] Emekci M, Navarro S, Donahaye E, Rindner M, Azrieli A (2004) Respiration of *Rhyzopertha dominica* (F.) at reduced oxygen concentrations. Journal of Stored Products Research 40: 27–38.

[20] Kaur R, Schlipalius DI, Collins PJ, Swain AJ, Rbert PR (2012) Inheritance and relative dominance, expressed as toxicity response and delayed development, of phosphine resistance in immature stages of *Rhyzopertha dominica* (F.) (Coleoptera: Bostrichidae). Journal of Stored Products Research 51: 74–80.

[21] Harshman LG, Schmid JL (1998) Evolution of starvation resistance in *Drosophila melanogaster*: aspects of metabolism and counter-impact selection. Evolution 52: 1679–1685. 28565325 10.1111/j.1558-5646.1998.tb02247.x

[22] Baldal EA, Brakefield PM, Zwaan BJ (2006) Multitrait evolution in lines of *Drosophila melanogaster* selected for increased starvation resistance: The role of metabolic rate and implications for the evolution of longevity. Evolution 60: 1435–1444. 16929660

[23] Koura A, El-Halfawy MA (1971) Tolerance of certain stored products insects to starvation. Agricultural Research Review 51: 35–36.

[24] Khan MA (1983) Effect of humidity on adults of 10 different species of stored product beetles. Zeitschrift fur Angewandte Entomologie 95: 217–227.

[25] Daglish GJ (2006) Survival and reproduction of *Tribolium castaneum* (Herbst), *Rhyzopertha dominica* (F.) and *Sitophilus oryzae* (L.) following periods of starvation. Journal of Stored Products Research 42: 328–338.

[26] Hinton HE (1942) Secondary sexual characters of *Tribolium* . Nature 149: 500–501.

[27] FAO (1975) Recommended methods for the detection and measurement of resistance of agricultural pests to pesticides. FAO plant protection bulletin. 105–118.

[28] GenStat 14 Committee (2011) GenStat for Windows (14 edition), VSN International Limited, Oxford, UK.

[29] Bloch Qazi MC, Herbeck JT, Lewis SM (1996) Mechanisms of sperm transfer and storage in the red flour beetle (Coleoptera: Tenebrionidae). Annals of the Entomological Society of America 89: 892–897.

[30] Ridley AW, Hereward JP, Daglish GJ, Raghu S, Collins PJ, et al (2011) The spatiotemporal dynamics of *Tribolium castaneum* (Herbst): adult flight and gene flow. Molecular Ecology 20: 1635–1646. 21375637 10.1111/j.1365-294X.2011.05049.x

[31] Trauner J, Buning J (2007) Germ-cell cluster formation in the telotrophic meroistic ovary of *Tribolium castaneum* (Coleoptera, Polyphaga, Tenebrionidae) and its implication on insect phylogeny. Development, Genes and Evolution 217: 13–27. 17123126 10.1007/s00427-006-0114-3

[32] Taher M, Cutkomp LK (1983) Effects of sublethal doses of DDT and three other insecticides on *Tribolium confusum* J. DU V. (Coleoptera: Tenebrionidae). Journal of Stored Products Research 19: 43–50.

[33] Wijayaratne LKW, Fields PG, Arthur FH (2012) Effect of methoprene on the progeny production of *Tribolium castaneum* (Coleoptera: Tenebrionidae). Pest Management Science 68: 217–224. 21770015 10.1002/ps.2247

[34] Espejo M, Orozco F (1966) Effect of removing or putting males to fecundated or virgin females, respectively, on the egg laying rate during a long period of time in *Tibolium castaneum* . Tribolium Information Bulletin 9: 90–84.

[35] Lilford K, Fulford GR, Schlipalius DI, Ridley AW (2009) Fumigation of stored-product insects - a two locus model of phosphine resistance. In: Anderssen RS, Braddock RD, Newham LTH, editors. 18th World IMACS COngress and MODSIM09 International Congress on Modeling and Simulation Cairns, Australia: Modelling and Simulation Society of Australia and New Zealand and International Association for Mathematics and Computers in Simulation pp. http://mssanz.org.au/modsim09/B01/lilford.pdf.

[36] Shi M, Collins PJ, Ridsdill-Smith J, Renton M (2012) Individual-based modelling of the efficacy of fumigation tactics to control lesser grain borer (*Rhyzopertha dominica*) in stored grain. Journal of Stored Products Research 51: 23–32.

